# Annotation and analysis of the mitochondrial genome of *Coniothyrium glycines*, causal agent of red leaf blotch of soybean, reveals an abundance of homing endonucleases

**DOI:** 10.1371/journal.pone.0207062

**Published:** 2018-11-07

**Authors:** Christine L. Stone, Reid D. Frederick, Paul W. Tooley, Douglas G. Luster, Brittany Campos, Richard A. Winegar, Ulrich Melcher, Jacqueline Fletcher, Trenna Blagden

**Affiliations:** 1 United States Department of Agriculture-Agricultural Research Service, Foreign Disease-Weed Science Research Unit, Fort Detrick, Maryland, United States of America; 2 MRIGlobal, Global Health Surveillance & Diagnostics, Palm Bay, Florida, United States of America; 3 Department of Biochemistry and Molecular Biology, Oklahoma State University, Stillwater, Oklahoma, United States of America; 4 National Institute for Microbial Forensics & Food and Agricultural Biosecurity, Department of Entomology & Plant Pathology, Oklahoma State University, Stillwater, Oklahoma, United States of America; Institut de Genetique et Microbiologie, FRANCE

## Abstract

*Coniothyrium glycines*, the causal agent of soybean red leaf blotch, is a USDA APHIS-listed Plant Pathogen Select Agent and potential threat to US agriculture. Sequencing of the *C*. *glycines* mt genome revealed a circular 98,533-bp molecule with a mean GC content of 29.01%. It contains twelve of the mitochondrial genes typically involved in oxidative phosphorylation (*atp*6, *cob*, *cox*1-3, *nad*1-6, and *nad*4L), one for a ribosomal protein (*rps*3), four for hypothetical proteins, one for each of the small and large subunit ribosomal RNAs (*rns* and *rnl*) and a set of 30 tRNAs. Genes were encoded on both DNA strands with *cox1* and *cox2* occurring as adjacent genes having no intergenic spacers. Likewise, *nad2* and *nad3* are adjacent with no intergenic spacers and *nad5* is immediately followed by *nad4L* with an overlap of one base. Thirty-two introns, comprising 54.1% of the total mt genome, were identified within eight protein-coding genes and the *rnl*. Eighteen of the introns contained putative intronic ORFs with either LAGLIDADG or GIY-YIG homing endonuclease motifs, and an additional eleven introns showed evidence of truncated or degenerate endonuclease motifs. One intron possessed a degenerate N-acetyl-transferase domain. *C*. *glycines* shares some conservation of gene order with other members of the Pleosporales, most notably *nad*6-*rnl*-*atp*6 and associated conserved tRNA clusters. Phylogenetic analysis of the twelve shared protein coding genes agrees with commonly accepted fungal taxonomy. *C*. *glycines* represents the second largest mt genome from a member of the Pleosporales sequenced to date. This research provides the first genomic information on *C*. *glycines*, which may provide targets for rapid diagnostic assays and population studies.

## Introduction

*Coniothyrium glycines* (R.B. Stewart) Verkely & Gruyter is a soilborne pathogen that infects soybeans and the perennial soybean, *Neonotonia wightii*, causing lesions on foliage, petioles, pods and stems and eventual defoliation and premature senescence [1]. *C*. *glycines* produces melanized sclerotia that can germinate to either form infectious mycelia or produce pycnidia that in turn produce infectious conidia. The pathogen is spread locally via rain/water splash and human or animal movement, which scatter sclerotia and conidia onto neighboring plants. Leaf drop of infected leaves delivers sclerotia and pycnidia to the soil where they serve as sources of secondary inoculum. Sclerotia may also remain in the soil and restart the cycle of infection in the next growing season. There is no evidence that the fungus is seed-borne, but spread might occur from infected plant debris mixed in with untreated seed or through movement of contaminated soil.

The disease red leaf blotch (RLB) occurs predominantly in central and southern Africa [2] and the incidence of the disease has increased concomitantly with increased soybean production in regions where the pathogen is found. Yield losses of up to 50% have been reported in Zambia and Zimbabwe [3][4]. While it does not currently occur within the United States, the ability of sclerotia to survive high temperatures and dry conditions suggest it could survive in soybean growing regions of the southern United States [5]. As a result, the Secretary of Agriculture has determined that *C*. *glycines* poses a significant risk to U.S. agriculture, and the pathogen is listed by USDA-APHIS as a Plant Pathogen Select Agent under 7 CFR, part 331 [6][7]. Additionally, while *C*. *glycines* has been found to naturally infect only soybean and *N*. *wightii*, there is no evidence as to the pathogen’s potential ability to infect other leguminous species, such as cultivated peanut and native, wild legumes that occur in the USA.

In the early stages of disease development, RLB may not be readily distinguished from other foliar soybean diseases such as *Alternaria* leaf spot, brown spot, or target spot. Current methods to identify *C*. *glycines* require time-consuming examination of morphological characteristics and temperature requirements. No molecular diagnostic assay currently exists to identify *C*. *glycines*. The examination of genomic sequences such as the mtDNA may provide targets for the development of diagnostic tools and also may provide insight into the mechanisms of disease resistance.

Phylogenetic analysis of the mtDNA will also be useful to clarify the taxonomy of this fungus. RLB was first observed on soybean in Ethiopia in 1955 and, based on the morphology of the pycnidial state, the causal fungus was identified as *Pyrenochaeta glycines* [8]. In 1964, *Dactuliophora glycines* was described as the cause of a leaf spot disease[9], and was subsequently identified as the sclerotial state of *P*. *glycines* [10]. Hartman and Sinclair [1] established the genus *Pyrenochaeta* to accommodate these synanamorphs. The fungus was re-classified as *Phoma glycinicola* in 2002 based on morphological characteristics[11][12], and most recently was again re-classified as *Coniothyrium glycines* (R.B. Stewart) Verkely & Gruyter based on sequence analysis of regions of the ITS, SSU, LSU [13]. The mt genomes of only eight other members of the class Dothidiomycete, which includes several economically important plant pathogens such as the wheat pathogen, *Stagonospora nodorum*, and wheat leaf blotch, *Zymoseptoria tritici (M*. *graminicola)*, can currently be found in GenBank. Six of these also share membership in the order Pleosporales with *C*. *glycines*. Comparison of the mt genome of *C*. *glycines* with the mt genome of these other eight fungi may help support or clarify the recent re-classification of *C*. *glycines*, as mitochondrial genomes are considered to be effective tools for evolutionary studies because they evolve independently of and at an accelerated rate from nuclear genomes [14][15][16].

This study provides the complete mitochondrial genome of a pathogenic fungus identified as a USDA-APHIS Plant Pathogen Select Agent due to its potential impact on soybean production. Previously, the only genomic data available were specific sequences used in phylogenetic analysis of *Phoma* and *Septoria* spp [13][17]. This sequence data may provide targets for the development of a rapid diagnostic assay and will help further clarify the evolving fungal taxonomy of the genus.

## Materials and methods

### Fungal isolate, library construction, and sequence assembly

*C*. *glycines-*infected leaves were collected from soybean at the Rattray Arnold Research Station, Harare, Zimbabwe in March 2005 and shipped to the USDA-ARS Foreign Disease-Weed Science Research Unit at Fort Detrick, MD under Animal and Plant Health Inspection Service permit. Isolate Pg-21 was recovered from the leaves and maintained on 20% V8-juice agar at 20°C in the dark. A 10% V8-juice broth was seeded with agar plugs containing mycelium of Pg-21 and grown for several weeks in the dark at 20°C without shaking. Tissue was collected through vacuum filtration onto Whatman No. 1 filter paper in a Buchner funnel. Total DNA was extracted using the DNeasy Plant Mini kit (Qiagen, Germantown, MD). Culture identification was confirmed through sequencing of ITS fragments.

The mt genome was sequenced as part of a whole genome sequencing project with Illumina sequence libraries prepared using Nextera XT. Whole genome 2×300 paired-end sequencing was performed using Illumina MiSeq instrument. Reads were filtered and trimmed using Trimmomatic v.0.32 [18]. The iMetAMOS pipeline v. 1.5[19] was used to optimize *de novo* assembly and perform quality checks. Elements of the pipeline include FastQC v. 0.10.0; Spades v. 3.1.1; IDBA v. 1.1.1; KmerGenie v. 1.6741; and QUAST v. 2.2 [20][21][22][23][24]. Resulting assemblies were polished using Pilon v. 1.8 [25]. Samtools v. 1.1[26] and BLAST were used to remove low coverage and contaminating contigs. Initial shotgun assembly produced 1431 contigs greater than 1kb in size, with a median size of 11kb and median depth of coverage of 274X. Contig 76 was identified as an outlier with a size of 98,482 bp and average depth of coverage of 1542X. Discontinuous MegaBLAST searches revealed homology with fungal mt genome sequences. Finishing of the mt sequence was performed using CLC Genomics Workbench Genome Finishing Module (Qiagen, Germantown, MD), mapping raw Illumina reads back to contig 76, correcting assembly errors, and extending the contig ends.

### Sequence annotation

The MFannot tool (http://megasun.bch.umontreal.ca/cgi-bin/mfannot/mfannotInterface.pl) was used to annotate the mt genome using genetic code 4 [27]. Annotation of open reading frames (ORFs) was reviewed and revised by BLAST homology searches against the NCBI protein database [28]. tRNAs were further evaluated against output from tRNAscan-SE[29], Dogma (Dual Organellar GenoMe Annotator)[30], and ARAGORN [31]. RNAweasel was used to classify identified introns as group I or group II introns [32]. Repeats were identified and analyzed with the Tandem Repeats Finder [33] and Palindrome and Einverted EMBOSS programs [34]. Codon usage for concatenated ORFs of twelve protein-coding genes was determined using the codon usage tool at http://www.bioinformatics.org/sms2/codon_usage.html with genetic code 4 [35]. The physical map of the *Coniothyrium* mtDNA was constructing using SnapGene Viewer (GSL Biotech; available at snapgene.com). The complete mt sequence of *C*. *glycines* isolate Pg-21 has been deposited in GenBank under the accession number MH337273.

### Comparative genomics

The complete mt genomes of the eight fungi belonging to the *Dothidiomycetes* were retrieved from GenBank (*Bipolaris cookei*, MF784482; *Didymella pinodes*, NC_029396; *Parastagonospora nodorum*, NC_009746; *Pithomyces chartarum*, KY792993; *Shiraia bambusicola*, NC_026869; *Stemphylium lycopersici*, KX453765; *Zasmidium cellare*, NC_030334; and *Zymoseptoria tritici*, NC_010222.) Mitochondrial gene content and gene order of *C*. *glycines* was compared visually to these eight fungi. Nineteen additional complete mt genomes were retrieved from GenBank for a comparison of general features, including size, GC content, core protein coding genes, rRNAs, and tRNAs, and the presence of introns.

### Phylogenetic analysis

Amino acid sequences of the twelve protein-coding genes shared in common among 25 fungal mt genomes were each aligned with MUSCLE from EMBL-EBI [36], and amino acids sharing low similarity were removed by Gblocks [37]. Sequences were concatenated using Seaview [38]. A maximum likelihood tree of aligned sequences was constructed with PhyML 3.0 using LG as the evolutionary model [39]. Branch support was assessed using the PhyML default of aLRT test (SH-Like).

## Results

### Gene content and genome organization

**Fig 1:**
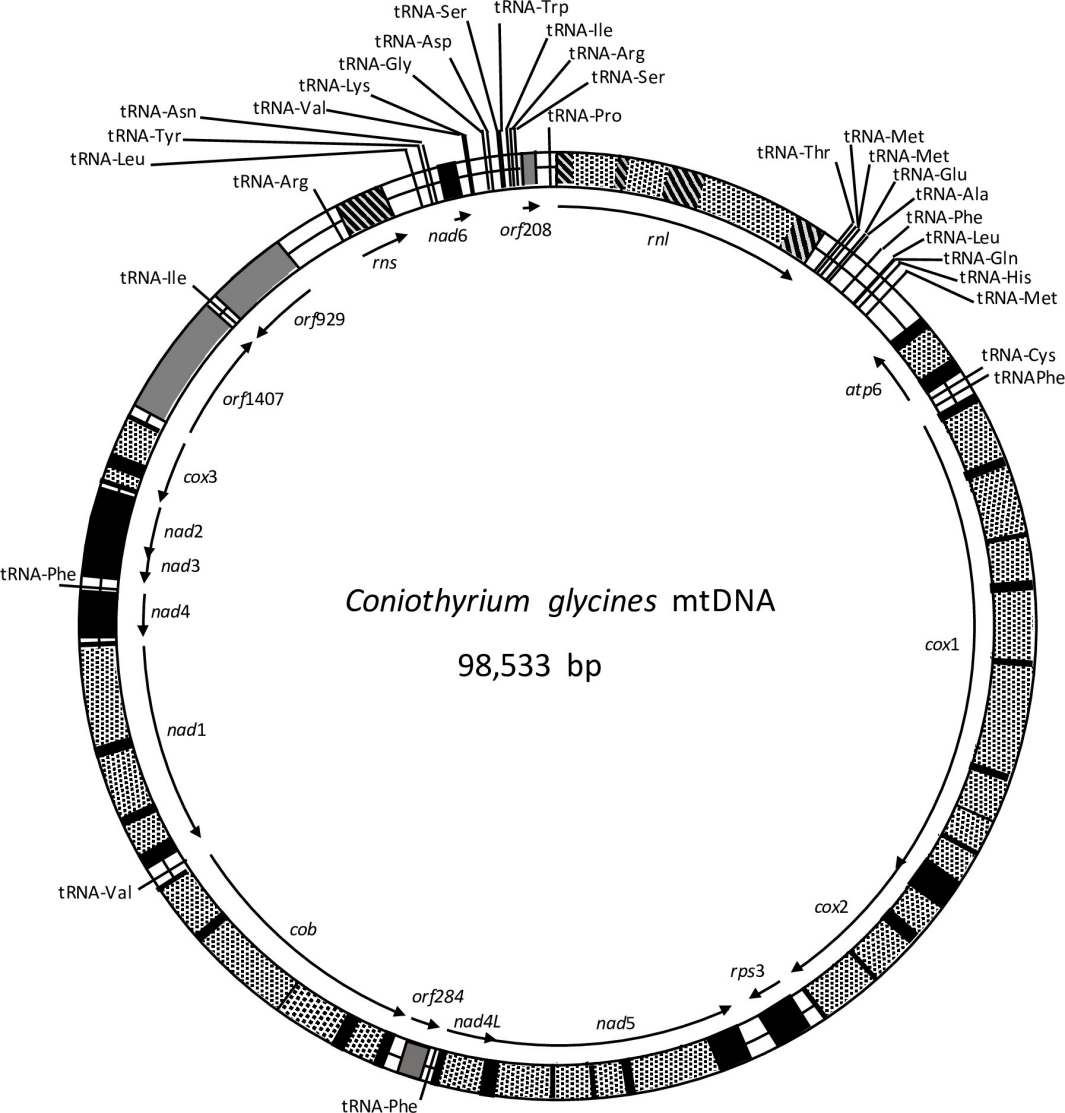
Circular mapping of the mitochondrial genome of *Coniothyrium glycines*. Black blocks, grey blocks, hatched blocks, stipled blocks, and bars show, respectively, protein-coding, orfs, rRNA, introns, and tRNA genes. Arrows indicate the direction of transcription.

The mt genome of *C*. *glycines* is a circular molecule with a length of 98,533 bp (Fig 1). The sequence is AT-rich with an overall G + C content of 29.01%, and 28.9% in the coding regions of the protein-coding genes. The RNA genes had a higher GC content of 35.1% while the intergenic spacers had a lower GC content of 24.8%.

Protein-coding genes of the mt genome included one gene encoding for ATP-synthase complex F0 subunit (*atp6*), three cytochrome oxidase subunits (*cox1*, *cox2*, *cox3*), seven nicotinamide adenine dinucleotide ubiquinone oxireductase subunits (*nad*1-6, *nad*4L), cytochrome b (*cob*), one ribosomal protein (*rps3*), and four hypothetical proteins (orf208, orf284, orf929, and orf1407) (Fig 1 and Table 1). The mt genome also encodes for small and large subunit ribosomal RNAs (*rns* and *rnl*) and 30 tRNAs (Fig 1 and Table 1). Genes were transcribed from both DNA strands. The *cox1* and *cox2* genes were adjacent to each other with no intergenic spacers. Similarly, *nad2* and *nad3* were adjacent with no intergenic spacers and *nad5* is immediately followed by *nad4L* with an overlap of one base (Fig 1 and Table 1).

**Table 1:** Gene content of the *Coniothyrium glycines* mitochondrial genome.

		Codon			
Genetic element	Location (nt)	Start	Stop	Size (nt)	Size (aa)
*rnl*	join: 1–642; 2155–2432; 3861–5119; 8396–9456			3240	
tRNA-Thr	9894–9964				
tRNA-Met	9989–10059				
tRNA-Met	10065–10137				
tRNA-Glu	10402–10474				
tRNA-Ala	10506–10577				
tRNA-Phe	11092–11164				
tRNA-Leu	11625–11707				
tRNA-Gln	11782–11853				
tRNA-His	11859–11931				
tRNA-Met	12192–12263				
*atp6*	join: 13717–14049; 15413–15853	ATG	TAG	774	257
tRNA-Cys	15948–16017				
tRNA-Phe	16300–16372				
cox1*	join: 16743–16954; 19031–19204; 21606–21712; 23153–23368; 24739–24749; 25790–25936; 27865–27867; 29552–29738; 31122–31189; 32251–32387; 33416–34214	ATG	*	2061	687
cox2*	join: 34215–34445; 35613–35976; 37630–37688; 39663–39761	TTA*	TAA	753	250
*rps*	40260–41591	ATG	TAA	1332	443
*nad5*	complement join: 42514–43641; 46586–46792; 47806–47952; 49229–49372; 51255–51680	ATG	TAA	2052	683
*nad4L*	complement, join: 51680–51709; 53178–53417	ATG	TAA	269	89
tRNA-Phe	complement 53450–53522				
orf284	complement, 53689–54543	ATG	TAA	855	284
*cob*	complement, join: 55085–55422; 56540–56872; 59027–59087; 62520–62747; 63283–63328; 64621–64775	ATG	TAG	1161	386
tRNA-Val	complement, 65040–65112				
*nad1*	complement, join: 65506–65987; 67161–67376; 69551–69827; 73205–73348	ATG	TAG	1119	372
*nad4*	complement, 73557–75110	ATG	TAA	1554	517
tRNA-Phe	complement, 75247–75319				
*nad3*	complement, 75575–76831	ATG	TAA	1257	418
*nad2*	complement, 76832–78580	ATG	TAA	1749	582
*cox3*	complement, join: 78714–78890; 79358–79780; 80968–81183	ATG	TAA	816	271
*orf1407/dpo*	81506–85729	ATA	TAA	4224	1407
*tRNA-Ile*	85795–85883				
*orf929/rpo*	complement, 85986–88775	ATG	TAA	2790	929
tRNA-Arg	90683–90753				
*rns*	91029–92648				
tRNA-Leu	93780–93862				
tRNA-Tyr	94127–94211				
tRNA-Asn	94286–94356				
*nad6*	94681–95268				
tRNA-Val	95545–95617				
tRNA-Lys	95650–95721				
tRNA-Gly	96262–96334				
tRNA-Asp	96337–96408				
tRNA-Ser	96658–96737				
tRNA-Trp	96846–96917				
tRNA-Ile	97024–97095				
tRNA-Arg	97100–97171				
tRNA-Ser	97271–97355				
orf208	97357–97983	ATG	TAA	627	208
tRNA-Pro	98372–98444				

*Putative polyprotein containing both *cox*1 & *cox*2.

Within the intergenic spacers, four open reading frames (orf208, orf284, orf929, and orf1407) were found (Fig 1 and Table 1). Putative functions could be assigned to three of the ORFs: orf1407 encodes a putative DNA polymerase type B, orf929 encodes a putative DNA-dependent RNA polymerase, and orf208 encodes a putative GIY-YIG endonuclease protein. All three showed similarity to relevant sequences in other fungi and possessed conserved domain motifs. Only orf284 contained no conserved motifs and could not be assigned a putative function, but showed similarity to hypothetical proteins from whole genome shotgun sequencing of *Bipolaris maydis* and *B*. *zeicola*. An additional GIY-YIG endonuclease motif was identified in the intergenic spacer between the *rnl* and *atp6*. This region showed similarity to endonucleases from other fungi, however no clear ORF could be identified suggesting that this may represent a degenerate endonuclease. Only 14.4% of the mt sequence is comprised of intergenic spacers.

Within the intergenic spacers, 10 perfect or near identical tandem repeats were identified ranging in size from 12–62 bp and with 2–5 copies (S1 Table). In addition, fifteen palindromes were identified ranging in size from 10–15 bp. A single inverted repeat of 30 bp was found.

### Introns

Introns made up 54.1% of the mt genome with a total of 32 introns identified within 8 of the protein-coding genes and the *rnl* (Fig 1 and Table 2). Thirty of the introns were classified as group I introns. One intron was classified as a group II intron (intron3 of the *rnl*) and one intron could not be definitively classified (intron2 of *cox*2). Eighteen of the identified introns were determined to contain putative intronic ORFs with either GIY-YIG or LAGLIDADG homing endonuclease (HE) motifs. An additional eleven introns showed evidence of truncated or degenerate HE motifs and one possessed degenerate N-acetyl-transferase domains. Only two introns had no identifiable ORFs and BLAST analysis revealed no homology in the NCBI protein database. All putative HEs showed significant similarity to those found in the mt genomes of other fungi and most were identified in other members of the Pezizomycotina subphylum. However, each was unique within *C*. *glycines*, showing no similarity to other intronic ORFs within the mt genome.

**Table 2:** Similarities of complete and truncated intron-encoded ORFs from the *Coniothyrium glycines* mtDNA to proteins in the non-redundant protein NCBI database (BLASTX <1e-05).

Gene	Intron	Conserved domain	E-value	Similarity	Accession
rnl	Intron 1	GIY-YIG endonuclease truncated	1.00E-68	*Bipolaris cookei*	YP_009445537.1
	Intron 2	GIY-YIG truncated	7.00E-117	*Sclerotinia borealis*	YP_009072317.1
	Intron 3	LAGLIDADG endonuclease	8.00E-86	*Chrysoporthe austroafricana*	YP_009262060.1
*atp*6	Intron 1	LAGLIDADG	0.0	*Bipolaris cookei*	YP_009445540.1
*cox*1	Intron 1	GIY-YIG	0.0	*Sclerotinia borealis*	YP_009072328.1
	Intron 2	LAGLIDADG truncated &	2.00E-127	*Bipolaris cookei*	YP_009445534.1
		GIY-YIG truncated	4.00E-46	*Chrysoporthe deuterocubensis*	YP_009262077.1
	Intron 3	GIY-YIG	0.0	*Bipolaris cookei*	YP_009445533.1
	Intron 4	LAGLIDADG	0.0	*Bipolaris cookei*	YP_009445530.1
	Intron 5	LAGLIDADG	7.00E-157	*Pyronema omphalodes*	YP_009240548.1
	Intron 6	LAGLIDADG truncated	6.00E-74	*Wickerhamomyces pijperi*	YP_008475104.1
	Intron 7	LAGLIDADG truncated &	1.00E-84	*Juglanconis oblonga*	ATI20220.1
		rps3/HE-like fusion protein	7e-33	*Sporothrix sp*.	ACV41149.1
	Intron 8	GIY-YIG	0.0	*Juglanconis oblonga*	ATI20221.1
	Intron 9	LAGLIDADG	0.0	*Bipolaris cookei*	YP_009445524.1
	Intron 10	GIY-YIG	2.00E-115	*Bipolaris cookei*	YP_009445523.1
*cox*2	Intron 1	GIY-YIG	2.00E-98	*Pestalotiopsis fici*	AFP72251.1
	Intron 2	GIY-YIG	2.00E-162	*Juglanconis juglandina*	ATI20502.1
	Intron 3	GIY-YIG	0.0	*Fusarium pseudograminearum*	CDL73109.1
*nad*5	Intron 1	LAGLIDADG	1.00E-180	*Chrysoporthe deuterocubensis*	YP_009262101.1
	Intron 2	LAGLIDADG	4.00E-142	*Bipolaris cookei*	YP_009445559.1
	Intron 3	LAGLIDADG	0.0	*Bipolaris cookei*	YP_009445560.1
	Intron 4	LAGLIDADG truncated	2.00E-130	*Annulohypoxylon stygium*	YP_008964963.1
*nad*4L	Intron 1	LAGLIDADG	8.00E-173	*Sclerotinia sclerotiorum*	YP_009389052.1
*cob*	Intron 1	LAGLIDADG	3.00E-26	*Fusarium culmorum*	YP_009136823.1
	Intron 2	-	-	*-*	-
	Intron 3	n-acetyl-transferase truncated	2.00E-94	*Stemphylium lycopersici*	KNG52863.1
	Intron 4	GIY-YIG truncated &	2E-41	*Sclerotinia borealis*	YP_009072335.1
		LAGLIDADG truncated	2E-133	*Cryphonectria parasitica*	AMX22249.1
	Intron 5	LAGLIDADG truncated	2.00E-122	*Podospora curvicolla*	CAB72448.1
*nad*1	Intron 1	GIY-YIG truncated &	5E-133	*Chrysoporthe austroafricana*	YP_009262069.1
		LAGLIDADG	0.0	*Bipolaris cookei*	YP_009445498.1
	Intron 2	LAGLIDADG	0.0	*Juglanconis oblonga*	ATI20217.1
	Intron 3	GIY-YIG truncated	2.00E-33	*Verticillium sp*.	ABU24266.1
*cox*3	Intron 1	LAGLIDADG truncated	4.00E-150	*Botrytis cinerea*	AGN49000.1
	Intron 2	-	-	*-*	-

A dash indicates no significant similarity of the intron sequence to any entries in the NCBI database.

The *cox*1 gene was the most common site for intron insertion, possessing ten of the 32 identified introns. Each of the ten introns also possessed either complete or degenerative putative HEs. Of these ten, only five were found to have high sequence identity to annotated introns found in the same location in the *cox*1 gene of the other Pleosporales. However, no other member of the Pleosporales possessed all five introns in common. The GIY-YIG HE of intron1 of *cox*1 showed 87% and 88% nucleotide identity to the corresponding introns of *D*. *pinodes* and *P*. *chartarum*, respectively. However, there was not a corresponding HE in the mt genomes of the other four Pleosporales species. Likewise, *cox*1 intron4, containing a LAGLIDADG HE, showed 88% nucleotide identity to the corresponding intron in *B*. *cookei*, but was found in no other Pleosporales species. The remaining five introns showed varying degrees of identity with introns from the mt genomes of more distantly related fungi, such as intron8 which showed 85% nucleotide identity with an intron from the corresponding location in *Sclerotinia sclerotiorum* (S2 Table).

The 2041-bp intron2 of *cox*1 has two regions with partial LAGLIDADG HE domains that showed 95–97% nucleotide identity with the 1208bp intron that occurs in the same position in the *cox*1 gene of *D*. *pinodes*. However, the central 1200 bp region of *cox*1 intron2 possessed a truncated GIY-YIG HE domain with no significant nucleotide similarity to any other fungus (S2 Table). This central region does show amino acid identity with a GIY-YIG HE located within an intron from the *cob* gene of the more distantly-related *Chrysoporthe deutercubensis* (Table 2).

While most introns showed nucleotide identity with introns inserted into the same gene in other fungi, *nad*4L intron1 shared identity with free standing orfs in *S*. *sclerotiorum* and *P*. *nodorum*. One intron, *nad*1 intron2, showed no nucleotide identity with other species from the Ascomycota, but rather showed identity with introns from two members of the Basidiomycota. This intron showed identity with an intron from the *nad*1 gene of *Moniliophthora roreri* and an intron from the *cox*1 gene of *Fomitopsis palustris*.

### Codon usage and tRNA genes

Codon usage, summarized in S3 Table, shows a bias towards AT-rich codons, which reflects the high AT content of the *C*. *glycines* mt genome. Most protein coding genes start with the canonical translation initiation codon ATG with the exception of *cox*2 and *orf*1407, which appear to utilize UUA and AUA start codons, respectively. The preferred stop codon in the mt genome was TAA, occurring in 12 genes. The alternative stop codon TAG occurs in 3 genes. A traditional stop codon could not be identified for *cox*1. This absence, combined with the location of *cox*1 adjacent to *cox*2 with no intergenic spacers, suggested the possibility of a fused *cox*1-*cox*2 polyprotein rather than two separate proteins. Thirty tRNAs were identified and twenty of them occurred in two large clusters around the *rnl*, while five occurred singly between mt genes (Fig 1). The tRNAs occurred on both DNA strands.

### Comparative genomics and phylogenetic analysis

**Fig 2:**
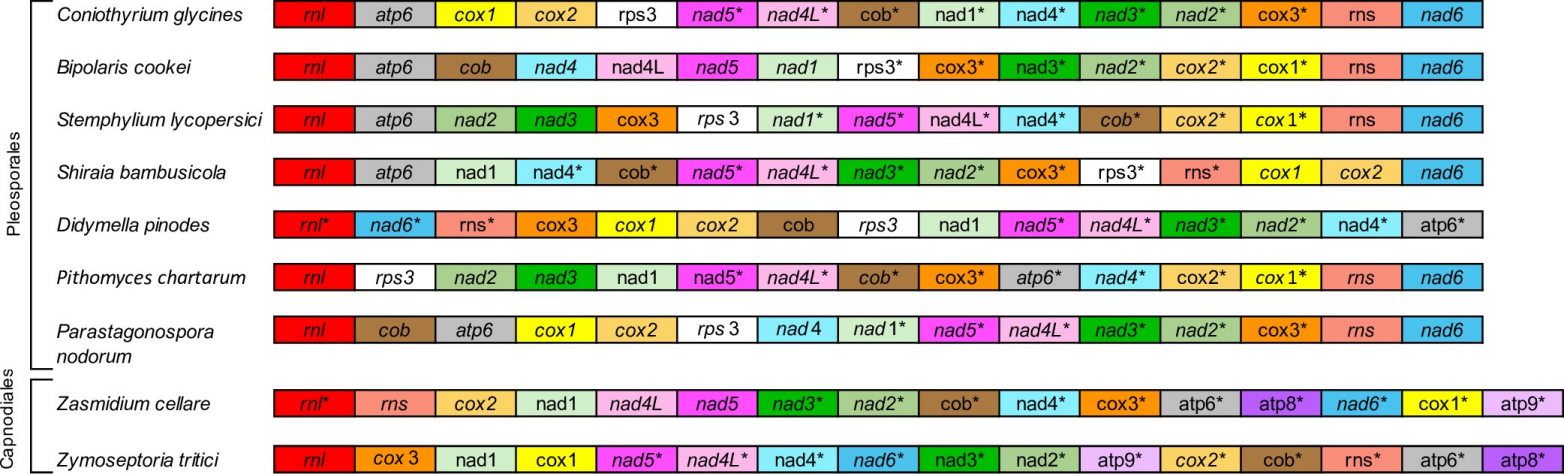
Mitochondrial genome rearrangements among Dothidiomycetes. Asterisk (*) indicates reverse direction of transcription. Each gene is assigned a separate color. Gene order was obtained from GenBank: *Bipolaris cookei* (MF784482), *Didymella pinodes* (NC_029396), *Parastagonospora nodorum* (NC_009746), *Pithomyces chartarum* (KY792993), *Shiraia bambusicola* (NC_026869), *Stemphylium lycopersici* (KX453765), *Zasmidium cellare* (NC_030334), and *Zymoseptoria tritici* (NC_010222).

Comparison of the mt genome of *C*. *glycines* with those from eight other members of the Dothidiomycetes revealed that in all nine species genes are encoded on both mtDNA strands. Comparison also found some conservation of gene order, most notably within the Order Pleosporales (Fig 2). In all nine species, *nad*4L and *nad*5 were adjacent, and in all but *P*. *nodorum* there are no intergenic spacers but rather a one base pair overlap between the two genes. Within *C*. *glycines* and the six members of the Pleosporales, *cox*1 and *cox*2 were also adjacent with no intergenic spacers. Three members of the Pleosporales possess a conserved gene block of *nad*5, *nad*4L, *nad*3, and *nad*2. *C*. *glycines* shows the same gene order, however the block is disrupted by insertion of *cob*, *nad*1, and *nad*4 between *nad*4L and *nad*3. *C*. *glycines* and the other Pleosporales species also lack the *atp*8 and *atp*9 genes which are typically found in fungal mt genomes, while both Capnodiales species possess both genes.

All nine species also exhibit large clusters of tRNA genes around the *rnl*, and within the Pleosporales tRNA order is maintained as well. The conservation of gene and tRNA order is expanded among the Pleosporales, with six of the seven possessing a *nad*6-*rnl*-*atp*6 gene block with associated conserved tRNA cluster patterns (Table 3). *P*. *chartarum* possesses a similar gene block and tRNA cluster pattern, but the *atp*6 is displaced relative to the other Pleosporales. This conservation of tRNA gene order is carried to a lesser extent to the Capnodiales.

**Table 3:** Comparison of conserved gene and tRNA cluster patterns flanking the rnl in *Coniothyrium glycines* and other Dothidiomycetes^a^.

Species	Order	Family	tRNA and gene order^b^	Accession
*Coniothyrium glycines*	Pleosporales	Coniothyriaceae	LYN-*nad*6-VKGDSWIRSP-*rnl*-TMM-EAFLQHM—*atp*6	MH337273
*Bipolaris cookei*	Pleosporales	Pleosporaceae	LYN-*nad*6-VKGDSWIRSP-*rnl*-TMM-EAFLQHML-*atp*6	MF784482
*Pithomyces chartarum*	Pleosporales	Pleosporaceae	-YN-*nad*6-VKGDSWIRSP-*rnl*-TMMLEAFLQHM	KY792993
*Stemphylium lycopersici*	Pleosporales	Pleosporaceae	LY—*nad*6-VKGDSWIRSP-*rnl*-TMMEAFLQHMNL-*atp*6	KX453765
*Didymella pinodes*	Pleosporales	Didymellaceae	LYN-*nad*6-V—DSWIRSP-*rnl*-TM EAFLQHM—*atp*6	NC_029396
*Shiraia bambusicola*	Pleosporales	Pleosporales incertae sedis	—N-*nad*6-V-GDSWIRSP-*rnl*-TMMEAFLQHM—*atp*6	NC_026869
*Parastagonospora nodorum*	Pleosporales	Phaeosphaeriaceae	LYN-*nad*6-VKGDSWIRSP-*rnl*-TMMEAFLQHM—*atp*6	NC_009746
*Zasmidium cellare*	Capnodiales	Mycosphaerellaceae	————GDSWI-SA-*rnl*—-LEFLQHMV	NC_030334
*Zymoseptoria tritici*	Capnodiales	Mycosphaerellaceae	————GDSWI-SP-*rnl*-MLEAFLYQMHRM	NC_010222

^a^The tRNA gene order of included organisms is taken from GenBank sequences.

^b^Capital letters correspond to tRNA genes for: L, Leucine; Y, Tyrosine; N, Asparagine; V, Valine; K, Lysine; G, Glycine; D, Aspartic acid; S, Serine; W, Tryptophan; I, Isoleucine; R, Arginine; P, Proline; T, Threonine; M, Methionine; E, Glutamic acid; A, Alanine; F, Phenylalanine; L, Leucine; Q, Glutamine; H, Histidine.

Comparison of the mt genome of *C*. *glycines* and the other Dothidiomycetes with those of an additional 19 ascomycetous fungal species revealed several potentially distinguishing characteristics of this class. Of the 25 mt genomes compared, fifteen carry all genes on the same strand of DNA and an additional four mt genomes show the core coding genes encoded on the same strand with only tRNAs or hypothetical proteins encoded in the opposite direction (S4 Table). However, all nine members of the Dothidiomycetes contain genes distributed on both mtDNA strands. Also, while ribosomal protein S3 or S5 occurs within an intron of the *rnl* in 17 of the 25 species examined, among the Pleosporales *rps*3/*rps*5 occurs as a free standing ORF and the gene appears to be absent from the two Capnodiales species (Table 4). Additionally, while *atp*8 and *atp*9 are absent from the Pleosporales species, both are found in the other species with the exception of *Pseudogymnoascus pannorum* which lacks only *atp*9 (Table 4). The proximity of *cox*1 and *cox*2, also characteristic of the Pleosporales examined to date, is not apparent among the other ascomycetous species.

**Table 4:** A comparison of the general features of some completely sequenced fungal mitochondrial genomes^a^.

Species	Size (bp)	GC content (%)	Core coding genes^b^	ribosomal protein^c^	rRNAs	tRNAs	introns	Accession
*Arthroderma otae*	23943	24.2	14	rps5	2	25	1	NC_012832
*Aspergillus niger*	31103	27.0	14	rps5^d^	2	25	3	NC_007445
*Beauveria bassiana*	29961	27.2	14	rps3	2	25	3	NC_010652
*Bipolaris cookei*	135790	30.1	12	rps3	2e	30	40	MF784482
*Botryotinia fuckeliana*	82212	29.9	14	rps3^d^	2^e^	30	20	KC832409
*Cladophialophora bantiana*	26821	24.5	14	rps5	2	22	2	NC_030600
***Coniothyrium glycines***	98533	29.0	12	rps3	2	30	32	MH337273
*Didymella pinodes*	55973	29.5	12	rps3^d^	2	22	14	NC_029396
*Epichloe typhina*	84630	27.0	14	rps3^d^	2^e^	24	18	NC_032063
*Glarea lozoyensis*	45038	29.8	14	rps3	2	33	7	KF169905
*Hypocrea jecorina*	42130	27.2	14	rps5	2	25	9	NC_003388
*Lecanicillium saksenae*	25919	26.5	14	rps3	2	26	1	NC_028330
*Metarhizium anisopliae*	24673	28.4	14	rps3	2	24	1	NC_008068
*Parastagonospora nodorum*	49761	29.4	12	rps5	2	27	5	NC_009746
*Peltigera dolichorrhiza*	51156	26.8	14	rps3^d^	2	26	6	NC_031804
*Penicillium polonicum*	28192	25.6	14	rps3	2	27	1	NC_030172
*Phialocephala subalpina*	43742	28.0	14	rps3	2	27	0	NC_015789
*Pithomyces chartarum*	68926	28.6	12	rps3^d^	2e	26	13	KY792993
*Pseudogymnoascus pannorum*	26918	28.1	13	rps3	2	27	1	NC_027422
*Pyronema omphalodes*	191189	43.0	14	rps3	2	25	22	NC_029745
*Sclerotinia borealis*	203051	32.1	14	rps3	2	31	61	NC_025200
*Shiraia bambusicola*	39030	25.2	12	rps3	2	32	1	NC_026869
*Stemphylium lycopersici*	75911	29.6	12	rps3^d^	2	28	15	KX453765
*Talaromyces marneffei*	35438	25.0	14	rps5	2	28	10	NC_005256
*Trichophyton rubrum*	26985	23.5	14	rps5	2	25	1	NC_012824
*Verticillium dahliae*	27184	27.3	14	rps3	2	25	1	NC_008248
*Zasmidium cellare*	23743	27.8	14	-	2	25	0	NC_030334
*Zymoseptoria tritici*	43964	32.0	14	-	2	27	0	NC_010222

^a^ All fungi in this table have mt genomes with circular topology.

^b^ Refers to the 14 conserved protein coding genes typical of fungal mitochondrial genomes: 11 genes encoding subunits of respiratory chain complexes (*cob cox1*, *cox2*, *cox3*, *nad1*, *nad2*, *nad3*, *nad4*, *nad4L*, *nad5*, and *nad6*) and 3 ATP synthase subunits (*atp6*, *atp8* and *atp9*).

^c^ Ribosomal protein S3 or S5, when present, occurs as an intronic orf within the rnl of all above mt genomes with the exception of *C*. *glycines*, *D*. *pinodes*, *P*. *nodorum*, *P*. *subalpina*, *P*. *amphalodes*, *P*. *chartarum*, *S*. *bambusicola*, *and S*. *lycopersici*.

^d^ The ribosomal proteins S3 or S5 were not annotated in the available sequences, but were putatively identified by blastx analysis against the non-redundant protein database.

^e^ Ribosomal RNAs were not annotated in the available sequences, but were putatively identified by blastn analysis against the rnl and rns of other fungal mt genomes.

Several similarities across the species were revealed as well. The G+C content is consistent among all species, ranging from 23–32%, with the exception of *Pyronema omphalodes* with 43%, and all show some tRNA clustering around the *rnl*. In all but four species, *nad*4L and *nad*5 are adjacent with either no intergenic spacer or a single base pair overlap (S4 Table).

The size of the mt genome and the presence of introns varies across all species, ranging from 23743 bp in *Z*. *cellare* with no introns to 203051 bp in *Sclerotinia borealis* with 61 introns. In general, a larger number of introns is reflected in a larger genome size (Table 4). Among the Pleosporales, *S*. *bambusicola* has the smallest mt genome at 39030 bp, of which only 3.2% is comprised of the one intron identified.[40] *P*. *nodorum* (49761 bp) contains five introns, which make up 13% of the mtDNA [16], while *D*. *pinodes* (55973 bp) contains 14 introns, making up 26% of its mt genome size (NC_029396). Within *C*. *glycines*, the 32 identified introns comprised 54% of total mt genome size.

**Fig 3:**
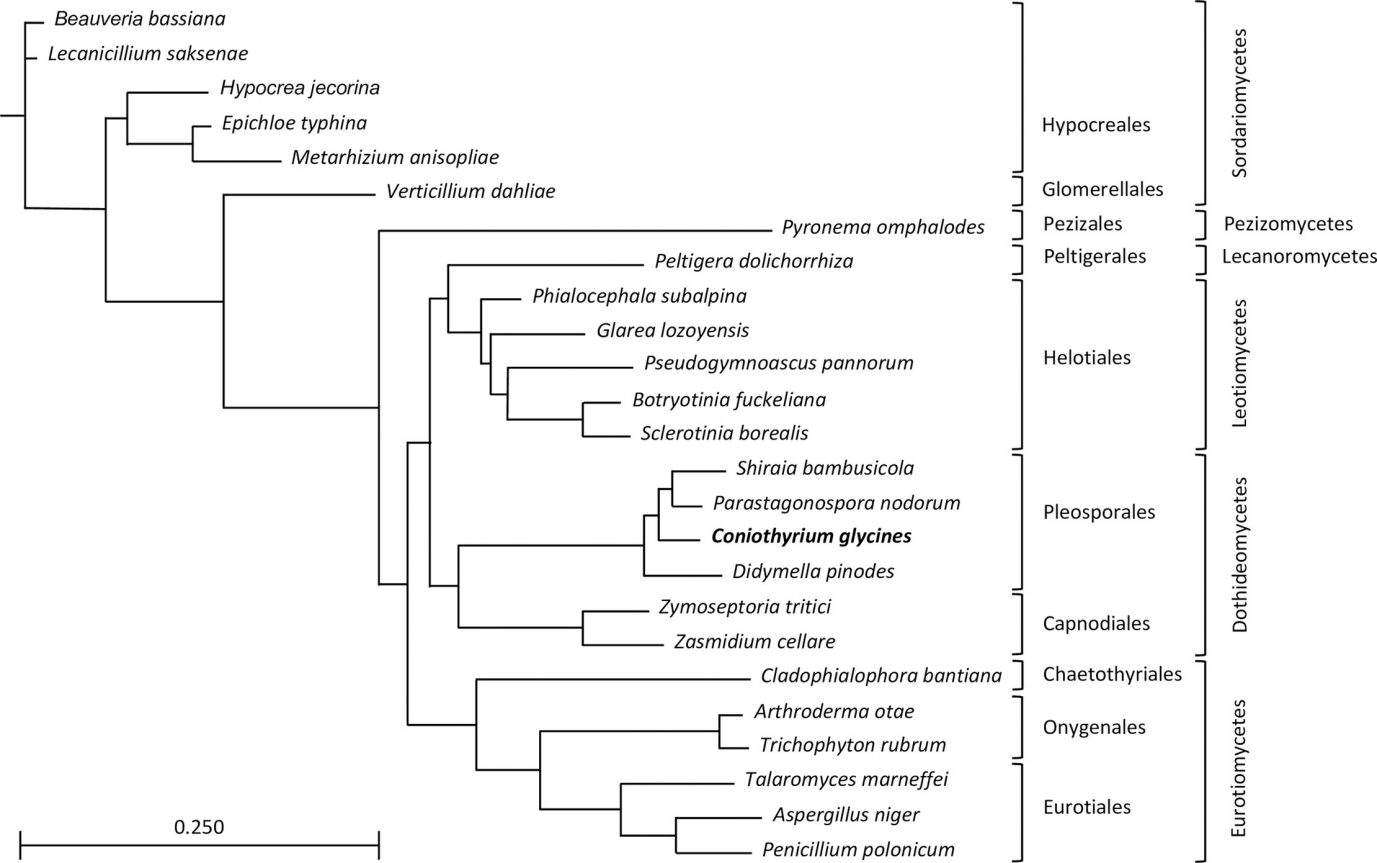
Phylogenetic tree constructed from unambiguously aligned portions of concatenated protein-coding sequences of twelve protein-coding genes shared in common among 25 fungal mt genomes. Topology shown was inferred with PhyML 3.0 using LG as the evolutionary model. Sequences were obtained from GenBank: *Arthroderma otae* (NC_012832); *Aspergillus niger* (NC_007445); *Beauveria bassiana* (NC_010652); *Botryotinia fuckeliana* (KC832409); *Cladophialophora bantiana* (NC_030600); *Didymella pinodes* (NC_029396): *Epichloe typhina* (NC_032063); *Glarea lozoyensis* (KF169905); *Hypocrea jecorina* (NC_003388); *Lecanicillium saksenae* (NC_028330); *Metarhizium anisopliae* (NC_008068); *Parastagonospora nodorum* (NC_009746); *Peltigera dolichorrhiza* (NC_031804); *Penicillium polonicum* (NC_030172); *Pseudogymnoascus pannorum* (NC_027422); *Pyronema omphalodes* (NC_029745); *Sclerotinia borealis* (NC_025200); *Shiraia bambusicola* (NC_026869); *Talaromyces marneffei* (NC_005256); *Trichophyton rubrum* (NC_012824); *Verticillium dahliae* (NC_008248); *Zasmidium cellare* (NC_030334); *Zymoseptoria tritici* (NC_010222); *Phialocephala subalpina* (NC_015789).

A phylogenetic tree was built with twelve protein-coding genes in common from 25 fungal species (Fig 3). This tree agrees with commonly accepted fungal taxonomy and supports the placement of *C*. *glycines* among the Pleosporales and recent reclassification to its own family, the Coniothyriaceae.[13]

## Discussion

This research provides the first genomic information on the USDA APHIS-listed Plant Pathogen Select Agent *C*. *glycines*; data which may provide targets for rapid diagnostic assays and population studies. Additionally, *C*. *glycines* represents the second largest mt genome from a member of the Pleosporales sequenced to date. Mitochondrial genome size among fungi varies greatly from the smallest, *Rozella allomyces*, at 12055 bp [41] to the largest, *Rhizoctonia solani*, at 235849 bp [42]. At 98,533 bp, *C*. *glycines* is of larger than average size and only 23 other currently available fungal mt genomes are larger. Among the fungi there is no correlation between mtDNA size and gene content.

The gene content of fungal mt genomes is largely conserved. However, it is notable that *C*. *glycines* lacked two of the core set of genes typical of fungal mt genomes: *atp*8 and *atp*9. These two genes were also absent from the mt genomes of other Pleosporales species [16][40]. While gene content may be conserved, gene order is not equally conserved and relative gene order varies both between and within major fungal phyla [43][44][45]. Alignment of the *C*. *glycines* mt genome with other members of the Dothidiomycetes identified a lack of synteny in gene order and gene orientation. However, limited conserved gene blocks were observed. The uninterrupted gene pairs of *nad*2-*nad*3 and *nad*4L-*nad*5 occurred in all nine Dothidiomycetes species, while the pairing of *cox*1-*cox*2 occurred only within all seven Pleosporales species and not the two Capnodiales species. Additionally, *nad*1-*nad*4 remain coupled in only three species from the Pleosporales. A conserved gene block *nad*2-*nad*3 and *nad*4L-*nad*5 was identified among three of the Pleosporales, but within the *C*. *glycines* mt genome this block is interrupted by three other genes. However, six of the seven Pleosporales species showed an *atp*6-*rnl*-*nad*6 conserved gene block, which included two large clusters of tRNAs on either side of the *rnl* in a relatively conserved pattern. Additionally, protein-coding and tRNA genes of *C*. *glycines* and the eight other Dothidiomycetes are encoded on both mtDNA strands, while the majority of ascomycetes species examined here have genes encoded on a single DNA strand. The pattern of gene order in mt genomes may provide a road map to trace the evolutionary route of fungal taxonomy. As additional species from the Dothidiomycetes, and the Pleosporales specifically, are analyzed, the additional mt signals will indicate if conserved gene blocks identified to date are characteristic of the Order Pleosporales and further help elucidate fungal taxonomy. Comparative genomics and phylogenetic analysis presented here supports the placement of *C*. *glycines* within the Pleosporales and its recent reclassification to its own family, the *Coniothyriaceae* [13].

With gene content being largely conserved, the size variation evident among fungal mt genomes is instead attributable to variations in the structure and size of intergenic spacers and the number and size of introns [46][47][48]. The larger than average mt genome size of *C*. *glycines* was attributed to the relatively high number of introns identified, with 32 introns comprising over half of the total mt genome size. This abundance of introns, most of which possess complete or degenerate HEs, may also provide valuable tools for the evaluation of evolutionary history and intron mobility [49][50][51][52][53][54]. While the *cox*1 gene is considered the most common insertion site for group I introns in fungal mt genomes, the number of introns inserted varies widely from zero in some fungi to the fourteen identified in *Podospora anserina* [55]. The present study of *C*. *glycines* found five of ten *cox*1 introns, which all possess either complete or truncated HE domains, shared high sequence identity with corresponding introns from the six other Pleosporales species annotated, suggesting common ancestral origin. However, it is notable that none of these five putative HEs occurred in all seven Pleosporales species. The remaining five *cox*1 introns showed varying degrees of identity with introns from the mt genomes of more distantly related fungi. For example, *cox*1 intron8 contained a GIY-YIG HE that showed 85% nucleotide identity with an intron from the corresponding location in *S*. *sclerotiorum* of the Helotiales and intron5, with its LAGLIDADG HE, shared 71% identity with an intron from *Lachancea mirantina*, a member of the Saccharomycotina subphylum (S2 Table). The similarity to HEs from more distantly related fungi suggest possible acquisition through horizontal transfer rather than retention from a common ancestor. Additional evidence of horizontal transfer comes from *nad*1 intron2 and its LAGLIDADG HE which showed no nucleotide identity with introns from other species of the Ascomycota, but rather showed identity with introns from two distantly related members of the Basidiomycota.

The examination of *cox*1 HEs also revealed evidence of multiple insertion events during the course of evolution. While *cox*1 intron2 possessed end regions with truncated LAGLIDADG domains and high nucleotide identity to a single orthologous intron from *D*. *pinodes*, the central region of this intron, with a truncated GIY-YIG domain, showed only amino acid similarity to an intron from the *cob* gene of the more distantly-related *C*. *deutercubensis*, suggesting the insertion of a new sequence into an already present HE.

It is difficult to determine the precise roles that intron retention, intron acquisition through horizontal transfer, and intron loss have played in constructing the *C*. *glycines* mt genome as it has been annotated here. The question remains if some fungal lineages possess a mechanism by which they accumulate and retain HEs while other fungal lineages appear to have lost all introns, and what that mechanism might be. However, this analysis of HEs does suggest that a complex pattern of insertions and horizontal transfers of introns are responsible for the relatively large mt genome size of *C*. *glycines*.

## Supporting information

S1 Table Repeat sequences in the *Coniothyrium glycines* mitochondrial genome. (DOCX)

S2 Table Sequence similarity betwen mt introns of *Coniothyrium glycines* and introns of other fungal mitochondrial genomes. (XLSX)

S3 Table Codon usage in twelve protein-coding mitochondrial genes of *Coniothyrium glycines*. (XLSX)

S4 Table Gene order of the fungal mt genomes used for comparative genomics and phylogenetic analysis. (XLSX)
