# Supplementary material for: Annotation and analysis of the mitochondrial genome of Coniothyrium glycines, causal agent of red leaf blotch of soybean, reveals an abundance of homing endonucleases
Source: PLoS One. 2018 Nov 7;13(11):e0207062. doi: 10.1371/journal.pone.0207062 (PMC6221350; doi:10.1371/journal.pone.0207062)
Supplement: S1 Table — (DOCX) [file pone.0207062.s001.docx]

**S1 Table. Repeat sequences in the *Coniothyrium glycines* mitochondrial genome.**

Tandem repeats

Period

Location size Consensus pattern Copy

12563-12682 56 TAGAGTATATAATGCACTTTTGGGTTATGGGTATAATAACTTTTGTTTTGAGATAT 2.1

12580-12668 28 TTTTGGGTTATGGGTATAATAACTTTTG 3.2

16204-16249 14 ATAGTAAATTATTA 3.3

75422-75547 62 TAAAAAGAAATAATCTTACCGGGATCTTATATCACTAAGTAAAACATTCACCTACAAAAGTTA 2.0

89132-89168 16 CATATAAATATGGTTA 2.3

89897-89952 19 TTTCTTTATCTTTTACTTA 2.9

94450-94056 23 TATTTAATCATACACATAAAATG 2.5

96536-96585 18 GTTATAGTTTAACATAC 2.8

98188-98275 18 TTCTATTTTTATAGATCT 4.9

98190-98292 54 CTATTTTTATAGATATTTCTATTTTTATAGATAGGTCTATTATTATATATCTTC 1.9

Palindromes

Location Size Sequence

10935-10944 10 taaaaaaaaa

10956-10947 attttttttt

13281-13290 10 aaaatatttt

13355-13346 ttttataaaa

16528-16538 11 taaaaagcata

16561-16551 atttttcgtat

40046-40055 10 aaaaatatct

40142-40133 tttttataga

53535-53544 10 cggggggggg

53584-53575 gccccccccc

64805-64814 10 ttttatttaa

64877-64868 aaaataaatt

64972-64981 10 atctatttat

65027-65018 tagataaata

73527-73538 12 attaaaaagtat

73551-73540 taatttttcata

75313-75322 10 atttcttttt

75432-75423 taaagaaaaa

90961-90971 11 attattatata

90984-90974 taataatatat

93191-93201 11 taaaaaaaaaa

93287-93277 atttttttttt

94446-94455 10 ttaatattta

94539-94530 aattataaat

94959-94968 10 attattaaca

95034-95025 taataattgt

95010-95019 10 tttttttaat

95092-95083 aaaaaaatta

96596-96610 15 tatttcattttaata

96636-96622 ataaagtaaaattat

Inverted repeats

Location Matches Sequence

10657-10686 25/30 tcttatagattttttttgaaaacaacaaaa

12074-12045 agaatttttaaaaaaaaactttgctgtttt
